# The Influence of Administration Route in the Comparison of Dosage Forms

**DOI:** 10.5812/atr.10032

**Published:** 2013-06-01

**Authors:** Tansel Comoglu

**Affiliations:** 1 Department of Pharmaceutical Technology, Faculty of Pharmacy, Ankara University, Ankara, Turkey

**Keywords:** Gabapentin, Diclofenac, Tonsillectomy

*Dear Editor,*

The article by Mogadam et al. (1) regarding the comparison of analgesic effect of gabapentin and diclofenac on pain after tonsillectomy was interesting, as it explained the comparison of analgesic efficacy results of preoperative gabapentin and diclofenac administration on postoperative pain in tonsillectomy. However, it is noted that one of the main factors which influences the rate of drug absorption is the route of administration. Absorption of the same molecule, taken at the same dose, by the same patient, but under a different administration route or a different dosage form (e.g. capsule or suppository) will not have the same kinetics of absorption, nor the same bioavailability. Therapeutic efficacy can only be achieved after the absorption of the active drug. In rectal administration, in general, it usually takes 5-30 minutes for the drug to be absorbed from rectum, whereas capsules must be first dissolved so as to release the drug powder. The effect of orally administered drugs is largely dependent on the rate of absorption from the gastrointestinal tract and absorption of drug takes place the whole length of the gastrointestinal tract (2). The authors mentioned the results of Biyik et al.’s paper and reported that both gabapentin and diclofenac had an effect on postoperative pain and in which gabapentin’s analgesic effect is found longer (3). I want to remind Mogadam et al. that in this study both gabapentin and diclofenac were administered in the same route; orally, so their effect was comparable. In my opinion, examination of two variables (different administration routes and two different active ingredients) at the same time makes the experimental design of the study difficult to compare. It may be of more interest examining the analgesic effect of gabapentin and diclofenac’s rectal administration on post-operative pain in patients undergoing tonsillectomy.
